# Clinical Course of Postoperative Spinal Epidural Hematoma After Microendoscopic Laminectomy for Lumbar Spinal Stenosis

**DOI:** 10.7759/cureus.106071

**Published:** 2026-03-29

**Authors:** Junichi Yokosuka, Yuichi Takano, Katsuhiko Ishibashi, Yasushi Inomata, Kenta Taniguchi, Kento Takebayashi, Hirohiko Inanami, Hiroki Iwai

**Affiliations:** 1 Department of Orthopaedics, Inanami Spine and Joint Hospital, Tokyo, JPN; 2 Department of Orthopaedics, Iwai Orthopaedic Hospital, Tokyo, JPN

**Keywords:** lower limb paresis, lscs, lss, lumbar spinal stenosis, mel, microendoscopic laminectomy, poseh, postoperative spinal epidural hematoma, pseh, surgical complication

## Abstract

Background

Postoperative spinal epidural hematoma (POSEH) is a known complication of spine surgery that occasionally requires reoperation because of severe pain or neurological deficits. However, few studies have examined the clinical course of POSEH after microendoscopic laminectomy (MEL) for lumbar spinal stenosis (LSS). This study investigated the incidence, clinical presentation, and neurological outcomes of POSEH requiring reoperation after MEL for LSS.

Methods

We retrospectively reviewed patients who underwent MEL for LSS at a single institution between January 2016 and December 2022. Patients with previous lumbar spine surgery were excluded. The diagnosis of POSEH and the indication for reoperation were based on clinical symptoms, with or without MRI findings, in which the hematoma was judged to be the primary cause of symptoms. Clinical characteristics and postoperative courses of patients with POSEH were analyzed.

Results

In total, 2289 MEL procedures were analyzed. Twenty-three patients (1.00%) developed POSEH requiring evacuation. More than half of the patients (56.5%) developed leg pain within four hours after surgery, and 78.3% developed symptoms by postoperative day one. Only one patient (4.3%) showed delayed onset (postoperative day four). Paresis occurred in five patients (0.22%), all within seven hours after surgery, and all experienced leg pain within four hours. Hematoma evacuation was performed on the day of surgery in all patients with paresis. Four patients recovered completely after reoperation, whereas one patient had mild residual weakness. The rate of permanent neurological deficit was 0.04%.

Conclusions

POSEH requiring reoperation after MEL for LSS occurred in 1.00% of cases. Paresis developed in 0.22% of patients, and permanent neurological deficit occurred in only 0.04%. Most symptomatic hematomas developed during the early postoperative period, and all patients who developed paresis experienced leg pain within four hours after surgery. Careful monitoring during the early postoperative hours may facilitate early detection and timely management of POSEH.

## Introduction

Postoperative spinal epidural hematoma (POSEH) is a common complication of spine surgery. Reoperation is required if an epidural hematoma causes severe pain or paresis, and the rate of reoperation for epidural hematoma after lumbar decompression surgery reportedly ranges between 0.46% and 2.2% [1-6]. Risk factors for POSEH are controversial [2,3,6-18]. POSEH may cause permanent motor deficits; however, motor deficits are less likely to be permanent if the time to reoperation is short [1,7,17,19,20]. Many studies on POSEH examined the cervical to lumbar spine collectively, and most considered open surgery; however, only a few studies of POSEH after microendoscopic laminectomy (MEL) for lumbar spinal stenosis (LSS) have been reported to date [5,8,21,22], and the characteristics of POSEH in endoscopic surgery are unknown. The present study investigated the reoperation rate, clinical symptoms, and the rate of permanent neurological deficits in patients with POSEH after MEL for LSS.

## Materials and methods

We retrospectively reviewed patients who underwent MEL for LSS at a single institution, Iwai Orthopaedic Hospital, between January 2016 and December 2022. Patients with a history of previous lumbar spinal surgery were excluded. This retrospective observational study was approved by the institutional review board of Iwai Orthopaedic Hospital. At our institution, MEL is routinely performed for all patients with LSS, and conventional open decompression is not employed.

All surgical procedures were performed by multiple surgeons using the METRx® microendoscopy system (Medtronic Sofamor Danek, Memphis, TN, USA). Antiplatelet and anticoagulant medications were discontinued before surgery according to an established institutional protocol. Depending on the patient’s symptoms and radiographic findings, either a unilateral (paramedian) approach or a midline spinous process-splitting approach was used. When intraoperative hemostasis was considered insufficient, the surgeon applied a gelatin-thrombin matrix sealant (Floseal®; Baxter International, Inc., Deerfield, IL, USA).

After decompression, one or more closed-suction drains were routinely placed in the epidural space. In cases of dural injury, closed non-suction drains were used. Drainage tubes were typically removed on postoperative day two and occasionally on postoperative day one. All reoperations were performed using a microendoscopic technique through the same skin incision as the initial surgery, and no cases required conversion to conventional open decompression.

Postoperative neurological symptoms, including leg pain and motor weakness, were routinely assessed by the attending surgeons and ward staff during the early postoperative period. Magnetic resonance imaging (MRI) was performed in patients who developed severe leg pain or paresis after surgery.

The diagnosis of POSEH and the indication for reoperation were based on clinical symptoms, with or without MRI findings, in which the hematoma was judged to be the primary cause of symptoms. In cases with MRI, the diagnosis was confirmed when the hematoma caused clear compression of the dural sac. In cases requiring immediate reoperation without preoperative MRI, a clinically significant hematoma causing neural compression was confirmed intraoperatively.

Patients with POSEH requiring surgical evacuation (POSEH group) were compared with those without POSEH (non-POSEH group). Differences between the POSEH and non-POSEH groups were evaluated using Student’s t-test and Fisher’s exact test. A P-value <0.05 was considered statistically significant.

## Results

During the study period, 2289 surgeries were performed, and 23 patients (1.00%) developed POSEH requiring evacuation. The POSEH group had an average age of 69.5 years, an average BMI of 23.2, and 65.2% were male, with no significant differences compared to the non-POSEH group (Table 1). The incidence rates of hematoma for one, two, three, and four decompression levels were 0.63% (10/1570), 1.45% (9/611), 4.60% (4/83), and 0/4, respectively. On a per-decompression level basis, these rates were 0.63%, 0.73%, and 1.53%, with no statistically significant differences observed among groups (P = 0.24, Fisher’s exact test).

**Table 1:** Demographic characteristics of the patients. Sex distribution was compared using Fisher's exact test, and differences in age and BMI were analyzed using Student’s t-test. POSEH, postoperative spinal epidural hematoma; BMI, body mass index.

		POSEH	Non-POSEH	t-value	P-value
Number of patients		23	2268		
Sex (male)		15 (65.2%)	1401 (61.2%)		0.825
Age	Mean (SD)	69.5 (10.3)	69.3 (10.4)	0.077	0.938
BMI	Mean (SD)	23.2 (2.6)	24.7 (3.7)	1.916	0.056

The clinical course of the 23 patients with POSEH is summarized in Table 2. A total of 13 cases (56.5%) presented with leg pain within four hours after surgery, 15 (65.2%) within 12 hours, and 18 (78.3%) by postoperative day one. Only one case (4.3%) exhibited a delayed onset (postoperative day four or later), on day four (number 23). Paresis occurred in five patients (0.22%, 5/2289; numbers 1, 4, 5, 10, and 11), all within seven hours after surgery. All five patients experienced leg pain within four hours. All five who developed paresis underwent hematoma evacuation on the same day as the initial surgery. Among the five patients with paresis, two recovered completely immediately after reoperation (numbers 1 and 5), and two recovered completely four days later (numbers 10 and 11). One patient (number 4) did not fully recover from right lower limb paresis; the manual muscle testing (MMT) score of the right tibialis anterior remained 4 at five years postoperatively, with no limitation in activities of daily living.

**Table 2:** Overview of postoperative spinal epidural hematoma cases. Patients with POSEH who underwent reoperation are listed in ascending order of the duration from the initial surgery to hematoma evacuation. BMI, body mass index; AP, antiplatelet therapy; PMH, previous medical history; GTMS, gelatin-thrombin matrix sealant; MMT, muscle manual testing; TA, tibialis anterior; CI, cerebral infarction; SAH, subarachnoid hemorrhage; DM, diabetes mellitus; BC, breast cancer; UIA, unruptured intracranial aneurysm; CC, colon cancer; PD, Parkinson’s disease; NA, not applicable.

No.	Age	Sex	BMI	AP	PMH	Number of decompression levels	Time of initial surgery (min)	Dural tear	GTMS	Onset of leg pain	Paresis (MMT of TA)	Onset of paresis	Duration from initial surgery to evacuation	Recovery from paresis
1	77	M	24.6	+	CI	1	100	-	+	<1 h	+ (2/2)	<1 h	1.7 h	Full immediately
2	70	M	23.8	-	-	3	97	-	-	<2 h	-	NA	2.1 h	NA
3	70	F	19.1	-	SAH	1	82	-	-	<1 h	-	NA	2.2 h	NA
4	71	F	25.0	-	-	2	72	-	-	<1 h	+ (2/3)	<1 h	2.6 h	Remain after 5 years
5	73	M	24.6	-	-	3	269	-	+	<3 h	+ (3/3)	<3 h	3.2 h	Full immediately
6	44	M	23.2	-	-	1	58	-	-	<2 h	-	NA	3.5 h	NA
7	80	M	21.6	-	DM	1	45	-	-	<2 h	-	NA	3.7 h	NA
8	50	M	23.4	-	-	2	247	+	+	<2 h	-	NA	4.1 h	NA
9	79	F	23.1	-	BC	1	48	-	-	<2 h	-	NA	4.4 h	NA
10	75	M	25.2	-	-	3	264	-	+	<4 h	+ (1/1)	<7 h	9.5 h	Full on day 4
11	73	F	21.0	-	-	2	112	-	-	<4 h	+ (1/1)	<7 h	9.6 h	Full on day 4
12	79	F	21.6	-	UIA	1	60	-	-	<5 h	-	NA	1 d	NA
13	78	M	23.5	-	CC	1	168	-	-	Day 1	-	NA	1 d	NA
14	57	M	22.0	-	-	2	112	-	-	3 h	-	NA	3 d	NA
15	51	F	19.9	-	-	1	41	-	-	<10 h	-	NA	3 d	NA
16	76	F	20.3	+	CI	2	162	-	+	<2 h	-	NA	5 d	NA
17	77	M	22.2	+	DM	1	68	-	-	Day 1	-	NA	6 d	NA
18	75	M	21.3	-	-	1	87	-	-	Day 1	-	NA	6 d	NA
19	79	F	22.3	+	CI	2	71	-	-	Day 2	-	NA	6 d	NA
20	72	M	24.6	-	DM	2	146	-	+	Day 2	-	NA	6 d	NA
21	61	M	21.6	-	PD	2	115	-	+	Day 3	-	NA	15 d	NA
22	68	M	28.1	-	DM	3	260	-	+	Day 3	-	NA	22 d	NA
23	63	M	30.3	-	-	2	152	-	-	Day 8	-	NA	30 d	NA

Among the 18 patients who presented with pain only, six underwent hematoma evacuation on the day of surgery, whereas eight underwent evacuation on postoperative day five or later. In three patients, including the case with delayed onset, hematoma evacuation was performed more than 10 days after symptom onset.

## Discussion

The incidence of POSEH after MEL for LSS was 1.00% in the present study, consistent with previous reports on lumbar decompression surgery. However, the timing of symptom onset differed from previous studies. More than 60% of patients developed symptoms on the day of surgery, and all but one did so within three days, whereas delayed onset has been reported more frequently in prior studies [1,4,23,24]. This difference may reflect the narrower subcutaneous space in endoscopic surgery than in conventional techniques. Age, BMI, and sex were not associated with POSEH. Although the number of operated levels was not significantly associated with POSEH, the incidence tended to increase with increasing numbers of operated levels. Multilevel procedures have been reported as a risk factor for postoperative epidural hematoma in open surgery [7,14]. Although endoscopic surgery treats each level in a separate operative field, no significant association was observed in the present study. Given the limited number of cases, particularly in multilevel procedures, this finding should be interpreted with caution.

The incidence of paresis caused by postoperative epidural hematoma was 0.22% (5/2289), and permanent paresis occurred in 0.04% (1/2289). Thus, the risk of permanent neurological deficit due to hematoma was extremely low. Even in the single patient with a persistent deficit, the weakness was mild, suggesting that postoperative hematoma after MEL rarely results in clinically significant neurological sequelae. Among the five patients who developed paresis, three did so within four hours after surgery (numbers 1, 4, and 5). The remaining two developed paresis seven hours after surgery, but had experienced leg pain within four hours (numbers 10 and 11). No patient whose leg pain developed later than four hours after surgery subsequently developed paresis. These findings suggest that the first four postoperative hours may be particularly important for patient monitoring. Previous studies involving the whole spine have similarly recommended close monitoring within the first four hours after surgery [7], and this recommendation may also apply to MEL for LSS.

One case (number 4) did not fully recover from paresis despite the short interval (2.6 hours) between the initial surgery and reoperation. In this patient, leg pain developed within one hour after surgery, suggesting rapid hematoma formation. Such an early onset of symptoms may reflect substantial bleeding, resulting in increased epidural pressure and severe neural compression, which may have contributed to the poor neurological outcome. Previous studies have indicated that a shorter time until reoperation [1,7,17,19,20] and less severe motor impairment before hematoma evacuation [18] were associated with better recovery. The present findings raise the possibility that a very early onset of paresis after surgery may be associated with a poorer neurological outcome. In cases in which paresis developed within a short time after surgery, urgent hematoma removal is desirable, and immediate surgical decompression should be considered.

Eighteen patients without paresis underwent reoperation, including 11 on the day of surgery; all had developed leg pain within four hours after surgery. Two additional patients (numbers 14 and 16) also developed leg pain within four hours but underwent reoperation on postoperative day one or later. Because reoperation in patients without paresis is generally a relative rather than urgent indication, the timing of reoperation varied among cases.

These findings may help clinicians determine the appropriate intensity and duration of postoperative monitoring after MEL for LSS, particularly during the early postoperative period.

This study has several limitations. We did not examine potential risk factors for postoperative epidural hematoma. However, the aim of this study was not to identify such risk factors but to clarify the clinical course of patients who required reoperation for hematoma. By analyzing these cases, we sought to determine how carefully patients should be monitored after surgery. The indication for reoperation may have varied among surgeons, particularly in patients without neurological deficits. While reoperation was mandatory in cases with paresis, the decision to perform reoperation in patients presenting with pain alone may have been influenced by the individual surgeon’s judgment. This variability may have introduced selection bias. The number of hematoma cases was relatively small (23 cases), and only five patients developed paresis; therefore, with a larger number of cases, some patients might develop pain and paresis more than four hours after surgery. Nevertheless, the total number of procedures exceeded 2000, providing meaningful clinical insight.

## Conclusions

POSEH requiring reoperation occurred in 1.00% of patients after MEL for LSS. Paresis developed in 0.22% of patients, and permanent neurological deficit occurred in only 0.04%. Most symptomatic hematomas developed in the early postoperative period, and all patients who developed paresis experienced leg pain within four hours after surgery. These findings suggest that careful monitoring during the early postoperative hours is important for the prompt detection and management of POSEH after MEL.
